# Determinants of the Differential Antizyme-Binding Affinity of Ornithine Decarboxylase

**DOI:** 10.1371/journal.pone.0026835

**Published:** 2011-11-03

**Authors:** Yen-Chin Liu, Den-Hua Hsu, Chi-Liang Huang, Yi-Liang Liu, Guang-Yaw Liu, Hui-Chih Hung

**Affiliations:** 1 Department of Life Sciences and Institute of Genomics and Bioinformatics, National Chung-Hsing University, Taichung, Taiwan; 2 Institute of Microbiology and Immunology, Chung Shan Medical University, Taichung, Taiwan; 3 Division of Allergy, Immunology, and Rheumatology, Chung Shan Medical University Hospital, Taichung, Taiwan; 4 Agricultural Biotechnology Center, National Chung Hsing University, Taichung, Taiwan; Aligarh Muslim University, India

## Abstract

Ornithine decarboxylase (ODC) is a ubiquitous enzyme that is conserved in all species from bacteria to humans. Mammalian ODC is degraded by the proteasome in a ubiquitin-independent manner by direct binding to the antizyme (AZ). In contrast, Trypanosoma brucei ODC has a low binding affinity toward AZ. In this study, we identified key amino acid residues that govern the differential AZ binding affinity of human and Trypanosoma brucei ODC. Multiple sequence alignments of the ODC putative AZ-binding site highlights several key amino acid residues that are different between the human and Trypanosoma brucei ODC protein sequences, including residue 119, 124,125, 129, 136, 137 and 140 (the numbers is for human ODC). We generated a septuple human ODC mutant protein where these seven bases were mutated to match the Trypanosoma brucei ODC protein sequence. The septuple mutant protein was much less sensitive to AZ inhibition compared to the WT protein, suggesting that these amino acid residues play a role in human ODC-AZ binding. Additional experiments with sextuple mutants suggest that residue 137 plays a direct role in AZ binding, and residues 119 and 140 play secondary roles in AZ binding. The dissociation constants were also calculated to quantify the affinity of the ODC-AZ binding interaction. The *K*_d_ value for the wild type ODC protein-AZ heterodimer ([ODC_WT]-AZ) is approximately 0.22 μM, while the *K*_d_ value for the septuple mutant-AZ heterodimer ([ODC_7M]-AZ) is approximately 12.4 μM. The greater than 50-fold increase in [ODC_7M]-AZ binding affinity shows that the ODC-7M enzyme has a much lower binding affinity toward AZ. For the mutant proteins ODC_7M(-Q119H) and ODC_7M(-V137D), the *K*_d_ was 1.4 and 1.2 μM, respectively. These affinities are 6-fold higher than the WT_ODC *K*_d_, which suggests that residues 119 and 137 play a role in AZ binding.

## Introduction

Ornithine decarboxylase (ODC, EC 4.1.1.17) is a pyridoxal 5′phosphate-dependent enzyme that catalyzes the decarboxylation of ornithine to putrescine [1], [2]. This reaction is the first and rate-limiting reaction in polyamine biosynthesis [3], [4], which is essential for eukaryotic cell growth and differentiation [5]–[7]. Polyamines and ODC play important roles in many biological functions, including the cell cycle, cellular proliferation, differentiation, apoptosis and embryonic development [8]–[17]. High levels of polyamines and ODC have also been associated with human disease and a variety of cancers [3], [18]–[23]. Because the concentration of ODC and polyamine is critical for cell proliferation [11], as well as during the development of neoplastic disease [24]–[28], ODC is considered to be an oncogenic enzyme. Regulation of ODC and polyamine levels is a current target for therapeutic studies involving numerous types of cancer [13], [29]–[33].

ODC activity *in vivo* is highly regulated through several pathways (reviewed in [3], [6], [18], [19]). For instance, the ODC protein has a short half-life and turns over very rapidly [34], [35]. A majority of proteins are degraded through ubiquitination, but ODC is degraded by the proteasome in a ubiquitin-independent manner via direct binding to the antizyme protein (AZ), which is regulated by polyamines [36]–[39]. AZ binds to ODC and promotes the dissociation of ODC homodimers and then forms the AZ-ODC heterodimer which is ultimately degraded by the 26S proteasome [3], [19], [40]–[42]. Several studies have shown that 37 residues in the C-terminus of ODC are important for degradation [41], [43], [44], and deletion of this region stabilizes ODC, even in the presence of AZ [45]. Moreover, additional studies have shown that residues 117–140 of ODC may play a role in AZ binding, which induces a conformational change in ODC that exposes the C-terminus and leads to recognition/degradation by the 26S proteasome [35], [36], [46].

The ODC protein circulates as a homodimer, and dimer formation is essential for enzyme activity [3], [47], [48]. The active site is located at the dimer interface, which is formed by the N-terminal domain of one subunit and the C-terminal domain of the second subunit [47]. Disruption of the dimer interface causes a loss of enzyme activity [42]. ODC can bind to AZ to form a heterodimer, and the AZ residues Glu-161, Glu-164 and Glu-165 seem to allow, through electrostatic interactions, the binding between of ODC to AZ to form a heterodimer [49]. The binding of AZ with ODC causes the ODC dimer to dissociate and thus inhibits ODC enzyme activity [3], [42].

AZ expression is regulated by cellular levels of polyamine [38]. The AZ mRNA transcript contains two overlapping open reading frames (ORFs). As the cellular concentration of polyamines increases, it induces a translational frame-shift of the AZ mRNA, which produces a longer functional AZ protein [19], [50]–[52]. Moreover, the cellular polyamines and polyamine transporter are regulated by AZ. AZ not only inhibits ODC activity to suppress polyamine production but also restrains polyamine uptake and stimulates polyamine excretion, thus controlling polyamine levels 38,53–55. Through this mechanism, ODC activity is down-regulated by AZ if polyamines are excessively generated by ODC.

ODC is a ubiquitous enzyme that is conserved in all species from bacteria to humans. The fatal human disease African sleeping sickness is caused by the protozoan *Trypanosoma brucei.* The disease is currently treated with an irreversible inhibitor, DFMO (DL-α-difluoromethylornithine), which inhibits the Trypanosoma brucei ODC enzyme (*t*ODC) [56]–[58]. *t*ODC is more stable than *h*ODC, and *t*ODC lacks the C-terminus that appear to be important for human ODC protein degradation [34], [59]. Furthermore, *t*ODC has a low binding affinity toward AZ and thus has a long half-life in the Trypanosoma brucei [34]. Mutation of mouse ODC residues 117–140 to match the *t*ODC sequence disrupts both AZ binding and *in vivo* regulation, suggesting that this sequence within mouse ODC is important for AZ binding [34]. In this paper, we identified several amino acid residues that influence human ODC (*h*ODC) binding to AZ. Sequence alignments of residues 117–140 of the *h*ODC and *t*ODC proteins show that there are seven non-conserved amino acid residues within this region (Table 1). We therefore mutated these seven amino acid residues in *h*ODC to match the *t*ODC sequence and subsequently examined the binding affinity of the mutant human ODC toward AZ.

**Table 1:** Amino acid residues at the putative AZ-binding site of human ODC (*h*ODC), Trypanosoma brucei ODC (*t*ODC) and human AZI (*h*AZI).

	*h*ODC	*t*ODC	*h*AZI		*h*ODC	*t*ODC	*h*AZI
Residue	*Non-conserved*			Residue	*Conserved*		
**119**	**Q**	**H**	**Q**	120	I	I	I
**124**	**A**	**R**	**A**	122	Y	Y	Y
**125**	**N**	**D**	**K**	123	A	A	A
**129**	**Q**	**D**	**N**	127	G	G	G
**136**	**E**	**V**	**E**	128	V	V	V
**137**	**V**	**D**	**I**	131	M	M	M
**140**	**M**	**E**	**K**	132	T	T	T
Residue	***Similar***			133	F	F	C
121	K	R	K	134	D	D	D
126	N	S	V	138	E	E	E
130	M	V	I	139	L	L	L
135	S	C	N				

## Results and Discussion

Previous structural studies of human ODC suggest that residues 117–140 may be the putative AZ-binding site [34], [60]. Mutation of residues 117–140 in mouse ODC to match the *t*ODC sequence resulted in decreased binding affinity toward AZ, implying that this region is important for AZ binding [34]. In this AZ-binding region of ODC, some amino acid residues are diverse and they may be the factors determining the differential AZ-binding affinity among these organisms. We aligned and compared the protein sequences of *h*ODC and *t*ODC and chose seven candidate residues to further study based on the charge dissimilarities and hydrophobicity (Table 1). The mutations that were made to the human ODC protein are Q119H, A124R, N125D, Q129D, E136V, V137D and M140E.

### Analysis of AZ inhibition of the *h*ODC septuple mutant

**Figure 1:**
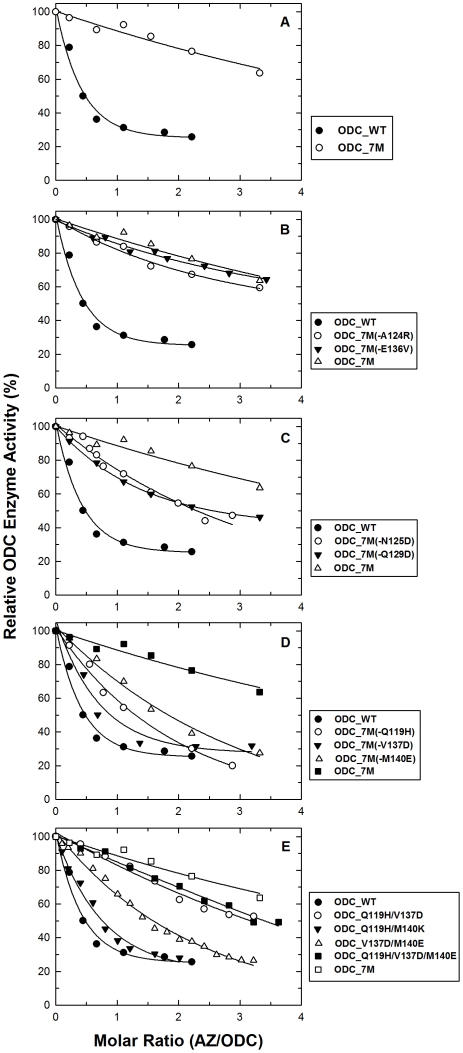
AZ inhibition of the wild type and ODC mutant proteins. **A:** inhibition plots for the ODC_WT and ODC_7M septuple enzymes; **B:** inhibition plots for the ODC_7M(-A124R) and ODC_7M(-E136V) sextuple mutant enzymes; **C:** inhibition plots for the ODC_7M(-N125D) and ODC_7M(-Q129D) sextuple mutant enzymes; **D:** inhibition plots for the ODC_7M(-Q119H), ODC_7M(-V137D) and ODC_7M(-M140E) sextuple mutant enzymes; **E:** inhibition plots for the ODC_Q119H/V137D, ODC_Q119H/M140E, ODC_V137D/M140E and ODC_Q119H/V137D/M140E mutant enzymes. The enzyme concentration was kept constant at 20 μg/mL, while the AZ concentration ranged from 0 to 28 μg/mL.

We initially simultaneously mutated all *seven* of these residues to generate a septuple mutant *h*ODC protein, which was named ODC_7M (ODC_Q119H/A124R/N125D/Q129D/E136V/V137D/M140E). For ODC_WT, the enzyme activity decreased with increasing concentrations of AZ. At a molar ratio of 2∶1 monomeric AZ to dimeric ODC, the ODC enzyme activity was approximately 30% (Figure 1A, closed circles). In contrast, the septuple ODC mutant was much less sensitive to AZ inhibition. At a molar ratio of 1∶1 AZ:ODC, the septuple ODC enzyme activity was approximately 90% (Figure 1A, open circles), indicating that these seven residues play a role in AZ binding and regulation of ODC enzyme activity.

### Analysis of AZ inhibition of *h*ODC sextuple mutants

By using ODC_7M as the template, we generated ODC mutant enzymes that had each possible combination of six point mutations. We created 7 sextuple mutants: ODC_7M(-Q119H), ODC_7M(-A124R), ODC_7M(-N125D), ODC_7M(-Q129D), ODC_7M(-E136V), ODC_7M(-V137D) and ODC_7M(-M140E). For ODC_7M(-Q119H), all of the residues were mutated except for Q119H (ODC_A124R/N125D/Q129D/E136V/V137D/M140E). The purpose of generating these sextuple mutants was to identify the essential amino acid residue(s) that govern ODC-AZ binding.

The inhibitory plots for these sextuple mutants are also shown in Figures 1. ODC_7M(-A124 R) and ODC_7M(-E136V) had a pattern of inhibition that was similar to ODC_7M (Figure 1B). These mutants were not significantly inhibited by AZ, which suggests that AZ is no longer binding and that residues 124 and 136 alone do not play a major role in AZ inhibition/binding. The ODC_7M(-N125D) and ODC_7M(-Q129D) enzymes were moderately resistant to AZ inhibition (Figure 1C), indicating that residues 125 and 129 may play a small role in AZ inhibition. The ODC_7M(-Q119H), ODC_7M(-V137D) and ODC_7M(-M140E) mutants had very little resistance to AZ inhibition (Figure 1D). This is especially obvious for ODC_7M(-V137D), which had an inhibition plot that is similar to ODC_WT, implying that residue 137 may be the most important amino acid residue involved in AZ binding and inhibition. The inhibitory plots of ODC_7M(-Q119H) and ODC_7M(-M140E) suggest that residues 119 and 140 may play secondary roles in human ODC-AZ binding.

### Analysis of AZ inhibition of *h*ODC double and triple mutants

To further confirm the significance of residues 119, 137 and 140 in AZ binding, double and triple *h*ODC mutants were created. The triple mutant ODC_Q119H/V137D/M140E had an inhibitory plot that is very similar to ODC_7M (Figure 1E, closed squares), again suggesting these three amino acid residues play a role in ODC binding to AZ. However, the ODC_Q119H/V137D double mutant was also resistant to AZ inhibition, similar to the triple mutant (Figure 1E, open circles), which suggests that residues 119 and 137 mediate AZ binding. Compared to the triple mutant ODC_Q119H/V137D/M140E, ODC_V137D/M140E displayed moderate AZ inhibition (Figure 1E, open triangles); absence of the Q119H mutation decreased the AZ inhibitory resistance. ODC_Q119H/M140E displayed minor AZ-inhibition resistance (Figure 1E, closed triangles), which again suggests that residue 137 plays a key role in *h*ODC binding to AZ.

### Dissociation constant of the *h*ODC-AZ heterodimer

**Figure 2:**
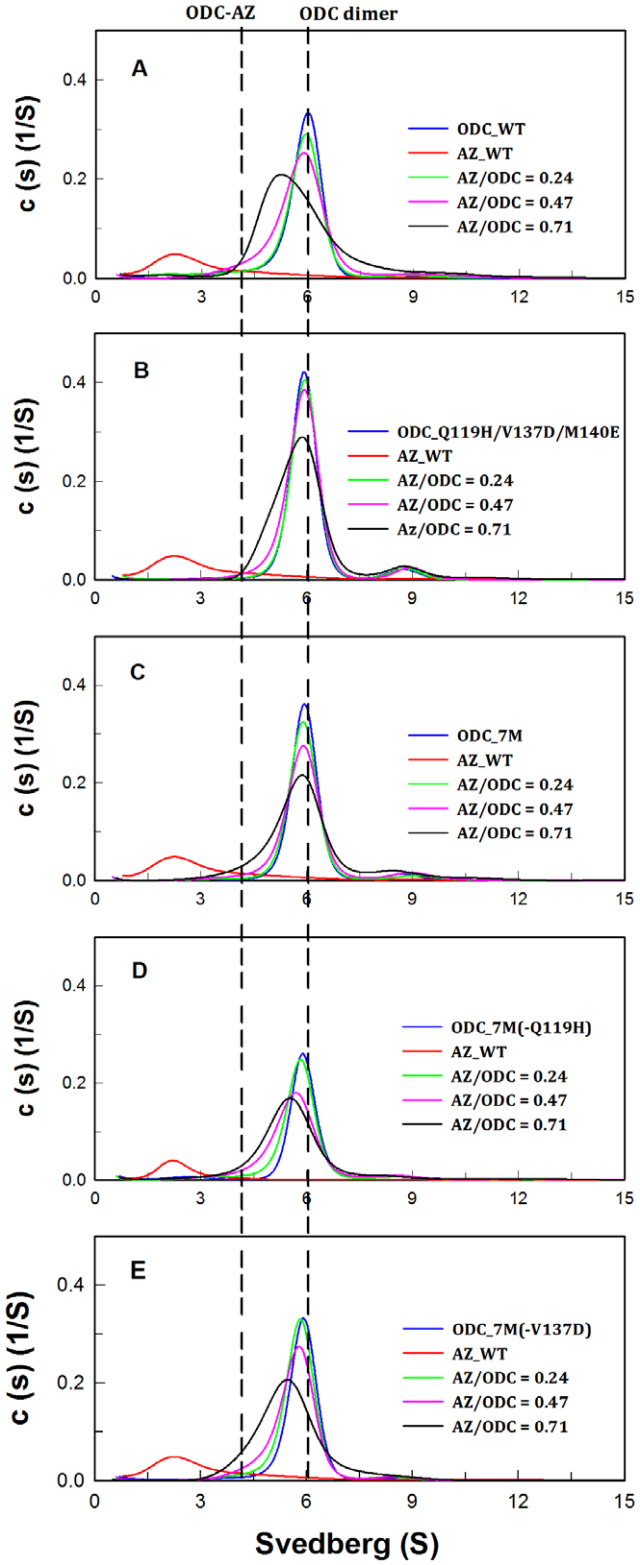
The continuous sedimentation coefficient distributions of human ODC mutant enzymes in the presence of AZ. The concentration of ODC was fixed at 0.3 mg/mL with an AZ concentration of 0.03, 0.06 or 0.09 mg/mL (the molar ratio of AZ/ODC was 0.24, 0.47 and 0.71, respectively). The sedimentation velocity data were globally fitted with the SEDPHAT program to calculate the ***K*** **_d_** values of the ODC-AZ heterodimer (Table 2). **A:** ODC_WT; **B:** ODC_Q119H/V137D/M140E; **C:** ODC_7M; **D:** ODC_7M(-Q119H); **E:** ODC_7M(-V137D).

To quantify the effect of *h*ODC mutations on the AZ binding affinity, dissociation constants (*K*_d_) were determined for the human WT and mutant ODC proteins (Table 2). Sedimentation velocity (SV) experiments with increasing AZ concentrations were performed, and the data were globally fitted to determine the dissociation constant of the ODC-AZ heterodimer (Table 2). Figure 2 shows the distribution plots of the WT and mutant ODC proteins. In the absence of AZ, ODC formed a stable dimer with an S-value of approximately 6; when AZ was present, ODC was dissociated. The ODC dimer peak shifted left and an ODC-AZ complex was formed, which had an S-value of approximately 4.1 (Figure 2A). The *K*_d_ value of the [ODC_WT]-AZ heterodimer is approximately 0.22 μM, while the [ODC_7M]-AZ heterodimer *K*_d_ is approximately 12.4 μM. The greater than 50-fold increase in the [ODC_WT]-AZ complex *K*_d_ indicates that the ODC-7M enzyme really has an extremely low binding affinity toward AZ. The triple mutant ODC_Q119H/V137D/M140E had an AZ-binding affinity that is similar to ODC_7M. The *K*_d_ for the [ODC_Q119H/V137D/M140E]-AZ heterodimer was about 9.9 μM, which is approximately 45-fold greater than the WT_ODC one. The *K*_d_ values for ODC_7M(-Q119H) and ODC_7M(-V137D) were 1.4 and 1.2 μM, respectively, and are 6-fold higher than the WT_ODC. These data again suggest that residues 119 and 137 play a key role in AZ binding.

**Table 2:** Dissociation constants of the human ODC-AZ complex.

*h*ODC-AZ complex	*K*_d_ (µM)*
[ODC_WT]-AZ	0.22±0.03
[ODC_Q119H/V137D/M140E]-AZ	9.95±0.36
[ODC_7M]-AZ	12.43±0.43
[ODC_7M(-Q119H)]-AZ	1.42±0.02
[ODC_7M(-V137D)]-AZ	1.20±0.02

*The *K*_d_ value was derived from global data fitting of the sedimentation velocity at three different protein concentrations of AZ (0.03–0.09 mg/mL, Fig. 2). The protein concentrations of human ODC were fixed at 0.3 mg/mL.

### Kinetic properties of human WT and mutant ODC enzymes

The kinetic parameters of the WT and mutant ODC enzymes were determined (Table S1). There were no obvious differences in *K*_m_ for ornithine substrate and PLP cofactor; additionally, the *k*_cat_ values for the WT and mutant enzymes were similar. These data suggest that the putative AZ-binding site mutations do not affect ODC enzymatic activity and may not induce a significant change in enzyme conformation.

### Charge effect in the putative AZ-binding site for differential AZ-binding affinity

Mutation of residues 117–140 in mouse ODC to mimic the Trypanosoma brucei ODC protein sequence abolished AZ binding [34], which suggests that several or all of these amino acid residues play a role in AZ binding. Our data clearly show that residues 137 and 119 in human ODC play key roles in AZ binding and influence the differential AZ-binding affinity of *h*ODC and *t*ODC. Mutation of Val137 to Asp and Gln119 to His (ODC_Q119H/V137D) may introduce a new charge to the AZ-binding element that may repel AZ and prevent binding. Whether Val137 and Gln119 in *h*ODC directly contact with AZ or simply stabilize the conformation in the AZ-binding site of *h*ODC cannot be clearly elucidated at this time. Crystal structural analysis of *h*ODC-AZ interaction may show the binding of these residues to their counterparts in AZ, however, this complex structure is not available.

Residues 125 and 129 are both aspartic acids, and residue 140 is glutamic acid in *t*ODC; however, in *h*ODC these positions are amino acid residues with neutral side chains (Table 1). Introduction of these negatively charged amino acid residues with the mutant *h*ODC decreases the binding affinity between ODC and AZ. The structural superimposition of *h*ODC and *t*ODC demonstrates a perfect overlapping with a RMSD value of 0.81, and the C_α_ positioning highlights the conserved AZ-binding element. The inability of *t*ODC to bind AZ and the weak binding affinity of the [ODC_7M]-AZ complex may result from the mutated charged amino acid residues at positions 119, 125, 129, 137 and 140 of the ODC enzyme.

### The putative AZ-binding residues of ODC

Sequence alignments at the putative AZ-binding site of *h*ODC, *t*ODC and human antizyme inhibitor (*h*AZI) is shown in Table 1. The AZI protein structure is homologous to ODC, and AZI has a higher binding affinity toward AZ [42]. Gln119 is highly conserved in all of the ODC and AZI enzymes, except in trypanosomes, suggesting that residue 119 may be crucial for the differential AZ-binding affinity between *h*ODC and *t*ODC but not between ODC and AZI. In contrast, residues 125 and 140 are not strictly conserved; in a majority of the ODC sequences, residue 125 is a neutral Asn or Ser and 140 is Met or Ser; however, in *t*ODC these residues are negatively charged Asp and Glu, respectively, while in AZI, they are a positively charged Lys. Our previous report has suggested that the differences in residues 125 and 140 between human ODC and AZI are responsible for the differential AZ-binding affinities [61]. Here we suggest that electrostatic effects are responsible for the differential AZ-binding affinities among *h*ODC, hAZI and tODC. Furthermore, residue 129 is Asn or Gln and residue 137 is Val or Ile in a majority of the ODC and AZI sequences, while these residues are Asp in *t*ODC. Based on the sequence comparisons and the mutagenesis analyses, we hypothesize that repulsive effect may occur in *t*ODC-AZ binding. The ODC_7M mutant, which has the *t*ODC sequence with Asp125, Asp129, Asp137 and Glu140, has a 50-fold smaller AZ-binding affinity than *h*ODC, suggesting the differences in charge properties of these residues play a key role in AZ-binding affinity of ODC.

## Materials and Methods

### Site-directed mutagenesis

Site-directed mutagenesis was performed with a QuikChange^TM^ kit (Stratagene, USA) to generate plasmids with the mutated human ODC (*h*ODC). Purified *h*ODC DNA was used as the template, which was PCR amplified with high-fidelity Pfu DNA polymerase and specific primers with the appropriate codon. The primers with the desired mutations were between 25 to 45 bases in length, which is necessary for specific binding to the template DNA. The sequence was amplified for 16–18 cycles. The PCR products were then treated with DpnI to cleave the wild-type *h*ODC template. The mutant PCR amplicons were cloned into a plasmid vector and transformed into XL-1 *E. coli.* The mutant plasmid DNA sequence was confirmed by autosequencing.

### Recombinant *h*ODC expression and purification

Human ODC or AZ was sub-cloned into the pQE30 vector (Qiagen, Hilden, Germany), which carries a N-terminal His6·Tag sequence for purification. This ampicillin-resistant vector was transformed into the JM109 strain of *Escherichia coli*. Recombinant protein expression was induced with 1.0 mM isopropyl-1-thio-β-D-galactoside (IPTG), and the cells were harvested at 25°C overnight. The recombinant protein was purified with a Ni-NTA Sepharose column (Sigma). The lysate-Ni-NTA mixture was washed with buffer that contained 10 mM imidazole, 500 mM NaCl and 30 mM Tris-HCl (pH 7.6). Recombinant ODC or AZ was eluted with buffer comprised of 250 mM imidazole, 500 mM NaCl, 2 mM β-mercaptoethanol and 30 mM Tris-HCl, pH 7.6. The purified ODC enzyme was buffer-exchanged and concentrated with 30 mM Tris-HCl (pH 7.6) and 2 mM β-mercaptoethanol with a 30 kDa molecular weight cutoff centrifugal filter device (Amicon Ultra-15, Millipore). The purified AZ protein was buffer-exchanged and concentrated with 250 mM NaCl, 30 mM Tris-HCl (pH 7.6) and 2 mM β-mercaptoethanol with a 10 kDa cutoff centrifugal filter device. The protein purity was verified by sodium dodecyl sulfate-polyacrylamide gel electrophoresis (SDS-PAGE), and the protein concentration was determined with the Bradford method [62].

### ODC enzyme assays

The decarboxylation of ornithine by ODC was measured with a CO_2_-L3K assay kit (DCL, Charlottetown, Canada) at 37°C. A continuous measurement of ODC enzyme activity was coupled with the decarboxylation of ornithine to the carboxylation of phosphoenolpyruvate (PEP) to form oxaloacetate (OAA), which becomes malate following NADH oxidation according to a previously published protocol [42]. The assay reaction mixture for spectrophotometry contained 30 mM Tris-HCl (pH 7.8), 10 mM ornithine, 0.2 mM PLP and 0.4 mL of CO_2_-L3K assay buffer containing 12.5 mM phosphoenolpyruvate, 0.4 unit/mL microbial phosphoenolpyruvate carboxylase, 4.1 units/mL mammalian malate dehydrogenase and 0.6 mM NADH analog in a final volume of 0.5 mL. ODC enzyme was added to initiate the reaction and the decrease of absorbance at 405 nm was continuously recorded with a Perkin-Elmer Lamba-25 spectrophotometer. In this coupled assay method, 1 mol of CO_2_ was formed and 1 mol of NADH analog was oxidized under the assay conditions. An absorption coefficient of 2,410 m^−1^ was used for the NADH analog in the calculations. All of the calculations were performed with the Sigma Plot 10.0 software program (Jandel, San Rafael, CA).

### Size-distribution analysis by analytical ultracentrifugation

Sedimentation velocity experiments were performed using a Beckman Optima XL-A analytical ultracentrifuge. Buffer (400 μl) and sample solutions (380 μl) were loaded into the double sector centerpiece individually and built up in a Beckman An-50 Ti rotor. The sedimentation velocity experiments were performed at 20°C with a rotor speed of 42,000 rpm. The protein samples were followed by continually monitoring UV absorbance at 280 nm with a time interval of 420 s and a step size of 0.002 cm. Multiple scans at different time points were fitted to a continuous size distribution model with the SEDFIT software [63], [64]. All of the size distributions were worked out at a confidence level of *p* = 0.95, a best fit average anhydrous frictional ratio (*f/f*_0_), and a resolution *N* of 250 sedimentation coefficients between 0.1 and 20.0 S.

To determine the dissociation constant (*K*_d_) for human ODC binding toward AZ, sedimentation velocity experiments were carried out at three different concentrations of AZ (0.03, 0.06 or 0.09 mg/mL) with a constant concentration of human ODC (0.3 mg/mL). All of the sedimentation data were globally fitted into the AB hetero-association model using the SEDPHAT program [65], [66] to calculate the *K*_d_ value for the ODC-AZ heterodimer. The partial specific volumes of the proteins, the solvent densities and the viscosity were calculated by the SEDNTERP program [67].

## Supporting Information

Table S1 **Kinetic parameters of the wild-type and mutant ODC enzymes.**
(DOC)
