# Supplementary material for: Determinants of the Differential Antizyme-Binding Affinity of Ornithine Decarboxylase
Source: PLoS One. 2011 Nov 3;6(11):e26835. doi: 10.1371/journal.pone.0026835 (PMC3207831; doi:10.1371/journal.pone.0026835)
Supplement: Table S1 — Kinetic parameters of the wild-type and mutant ODC enzymes. (DOC) [file pone.0026835.s001.doc]

Supplementary Table 1 Kinetic parameters of the wild-type and mutant ODC enzymes

| ODC | *K*_m,ornithine_ (mM) | *K*_m,PLP_ (μM) | *k*_cat_ (s^-1^) |
| --- | --- | --- | --- |
| ODC_WT | 0.29±0.06 | 0.9±0.19 | 2.15±0.14 |
| ODC_7M | 0.56±0.07 | 1.76±0.77 | 3.37±0.46 |
| ODC_7M(-Q119) | 0.48±0.03 | 0.88±0.05 | 4.01±0.08 |
| ODC_7M(-A124) | 0.58±0.02 | 0.77±0.11 | 4.47±0.23 |
| ODC_7M(-N125) | 0.42±0.01 | 0.77±0.06 | 3.88±0.11 |
| ODC_7M(-Q129) | 0.42±0.04 | 1.60±0.40 | 3.66±0.34 |
| ODC_7M(-E136) | 0.45±0.05 | 1.62±0.14 | 2.80±0.09 |
| ODC_7M(-V137) | 0.46±0.07 | 1.96±0.75 | 3.31±0.51 |
| ODC_7M(-M140) | 0.53±0.05 | 0.95+0.04 | 4.78+0.79 |
| ODC_Q119H/M140E | 0.40±0.02 | 0.99±0.16 | 3.24±0.15 |
| ODC_Q119H/V137D | 0.46±0.02 | 1.21±0.17 | 3.65±0.17 |
| ODC_V137D/M140E | 0.36±0.04 | 2.06±0.59 | 2.34±0.27 |
| ODC_Q119H/V137D/M140E | 0.53±0.02 | 1.76±0.77 | 3.31±0.46 |
